# Mechanism for XMRV neurotoxicity

**DOI:** 10.1186/1742-4690-8-S2-O18

**Published:** 2011-10-03

**Authors:** Andrew E Vaughan, Ramon Mendoza, Ramona Aranda, Jean-Luc Battini, A Dusty Miller

**Affiliations:** 1 Human Biology Division, Fred Hutchinson Cancer Research Center, HOC Fairview Avenue North, Seattle, WA 98109-1024, USA; 2 Retrovirus Replication and Pathogenesis Group, Institut de Génétigue Moléculaire de Montpellier, CNRS-UMSF, 1919 Route de Mende, 34293 Montpellier, Cedex 5, France

## 

Xenotropic murine leukemia virus-related virus (XMRV) has been found in a high percentage of humans with chronic fatigue syndrome (CFS). However, more recent studies have failed to confirm these results, and it now appears likely that the original findings were due to patient sample contamination. Because it initially appeared that XMRV was involved in CFS, we explored potential mechanisms of XMRV neurotoxicity that might underliethe neuromuscular pathology seen in CFS. Indeed, we found that XMRV infection induced apoptosis in SY5Y human neuroblastoma cells. We hypothesized that signaling through the cell-entry receptor for XMRV, the xenotropic and polytropic retrovirus receptor (Xpr1), mediated this toxicity. In support of this hypothesis, SY5Y cells expressing mouse Xpr1, which unlike human Xpr1 does not bind or promote entry of xenotropic retroviruses, were resistant to XMRV toxicity, even though XMRV could still infect these cells. Similarly, SY5Y cells expressing several XMRV binding-defective deletion mutants of human Xpr1 were resistant to XMRV toxicity. These results indicate that Xpr1 mediates the toxicity of XMRV.

Xpr1 is related the yeast Syg1 protein, which associates with the β subunit of the yeast G-protein. We found that human Xpr1 is also associated with the human Gβ subunit, and that over expression of mouse or human Xpr1 increased intracellular cAMP, a typical output of stimulatory G-protein signaling. Moreover, increasing the cAMP level in SY5Y cells by direct activation of adenylate cyclase protected the SY5Y cells from the toxic effects of XMRV and polytropic retrovirus infection. These results indicate that Xpr1 is a G-protein-coupled receptor (GPCR), and that xenotropic or polytropic retrovirus binding can disrupt the cAMP-mediated signaling function of Xpr1 leading to apoptosis of infected cells. In addition, we found that this pathway is responsible for the toxicity of the polytropic mink cell focus-forming (MCF) retrovirus in mink cells, the basis for the classic MCF focus assay.Xpr1 orthologs are widely distributed in animals, plants and unicellular organisms, but these proteins show no sequence similarity to known GPCRs. Some proteins with similarity to Xpr1 are involved in phosphate uptake into cells, but we found no role of Xpr1 in phosphate uptake or its regulation. Lastly, some polytropic retroviruses induce neurologic disease in mice, and we propose that alterations of Xpr1-mediated G-protein signalinglikely are responsible. However, because of recent results indicting that XMRV is not a human retrovirus, a role for XMRV in human disease is unlikely.

